# Development of a Treatment System of Water with Cr (VI) Through Models Using *E. crassipes* Biomass with Iron Chloride

**DOI:** 10.3390/toxics13030230

**Published:** 2025-03-20

**Authors:** Uriel Fernando Carreño Sayago, Vladimir Ballesteros Ballesteros, Angelica María Lozano

**Affiliations:** Faculty of Engineering and Basic Sciences, Fundación Universitaria los Libertadores, Bogotá 111221, Colombia; vladimir.ballesteros@libertadores.edu.co (V.B.B.); amlozanoa@libertadores.edu.co (A.M.L.)

**Keywords:** biomass, *E. crassipes*, isotherm, chromium

## Abstract

In the context of critical water quality issues, there is a pressing need for more pragmatic approaches to water research. Adsorbent biomass, derived from abundant and effective natural sources, holds considerable promise as a solution. *E. crassipes*, a type of plant biomass, has emerged as a particularly promising material due to its high adsorption capacity. When combined with iron chloride, this capacity is significantly enhanced, and the addition of EDTA is essential for the reuse of treated water. The economic viability of this material in water treatment has been thoroughly evaluated, and the project was developed with the aim of building treatment systems using *E. crassipes* biomass in conjunction with iron chloride. The development process involved the creation of a special material composed of 85% dried and ground *E. crassipes* and 15% iron chloride. The process was scaled up with the most effective biomass for treatment and subsequent elutions with EDTA. The outlet conditions, the quantity of pollutant removed, and the treated volume were established, and subsequently the extraparticle diffusion constant Kf, the intraparticle diffusion constant, and the characteristic isotherm were determined. The identification of the intraparticle diffusion model, Ks, was made possible by the results of the model, which indicated the specific route for the construction of a pilot-scale treatment system. The pilot-scale prototype was constructed using 1000 g of EC (2) of biomass (850 g of *E. crassipes* and 150 g of chloride of iron). The prototype developed in the present investigation could be used to treat effluents contaminated with heavy metals, especially chromium, and is an advanced environmental research project that contributes to the improvement of water quality.

## 1. Introduction

Water is a vital resource in research processes in environmental sciences around the world, and there is a need to care for, maintain, and treat this valuable resource well. However, few of these investigations are disruptive because the business sector continues to contaminate rivers, lagoons, wetlands and other bodies of water without any treatment process. Heavy metals, especially chromium (VI), are a major contaminant that generate serious consequences. This heavy metal is among the most noxious and has the potential to generate a wide range of environmental impacts, both immediately and over the long term, due to its mutagenic and carcinogenic properties. Exposure to this contaminant has been linked to respiratory, reproductive, and cardiovascular diseases in humans, as well as having other harmful effects on plants and animals [1,2,3,4]. Consequently, there is an urgent need to identify simple, practical and, above all, effective methods for treating effluents laden with various heavy metals. One such approach involves the utilization of waste plant biomass systems, which were found to be effective in treating water [5,6,7,8,9,10]. A notable example is *E. crassipes*, commonly referred to as water buckthorn, which is prevalent in wetlands and lagoons across Colombia. However, there is a paucity of concrete proposals concerning the sustainable management of this species [11,12,13,14,15,16,17]. *E. crassipes* has been employed in the removal of heavy metals at both laboratory and pilot scales [18,19,20,21,22,23,24]. In experimental processes, the adsorption capacity of biomass alone has been shown to be 15 mg/g for Cr (VI) [7] and approximately 45 mg/g for cellulose xanthate [5]. However, a treatment system of water has not yet been developed with this plant, and it is known from the design criteria that this plant is a good support material, but reinforcement is needed to increase the adsorption capacities, such as iron chloride. This reagent increases the adsorption capacities due to the deep oxidation of this biomass generating more active sites, such as hydroxyl groups (OH) [25,26,27,28,29]. The utilization of this reagent has facilitated the exploration of the benefits of heavy metal adsorption, yielding noteworthy outcomes. The utilization of iron chloride (Fe) (III) in the treatment of plant cellulose has been employed for the elimination of both organic and inorganic contaminants. (Fe) (III) reacts with hydratable hydroxyl cellulose, forming iron hydroxides (FeOOH), which are responsible for cation exchange with heavy metals. The metal ions enter the interior of *E. crassipes* with FeOOH, exchanging with protons of the hydroxyl groups [21]. The formation of iron hydroxides was characterized and evaluated, yielding 10 mg/g in As (III) [30]. Through the application of adsorption models, specialized designs were developed for the treatment of effluents, as evidenced by outer layer parameters (Kf) and inner layer (Ks) [31,32,33,34,35,36]. These parameters are utilized to determine the area of influence and the amount of biomass in conjunction with the design volume, adsorption capacities, and design flow rates. The experimental processes conducted at the laboratory scale have facilitated the acquisition of design variables, thereby enabling the identification of similarities in the adsorbent contact time and hydrodynamic characteristics between the adsorption systems. This, in turn, has paved the way for the development of systems at larger scales, akin to those developed at laboratory or pilot scales, and the modeling of the efficiency of larger column systems using vertical column systems [37,38,39,40,41,42]. However, a treatment system based on extraparticle and intraparticle diffusion models with *E. crassipes* biomass reinforced with iron chloride has not yet been constructed, including the benefits of EDTA elutions, in which this chemical reagent is a powerful eluent and serves to enable the biomass to be reused in chemical adsorption processes [43,44,45,46,47,48,49,50]. Consequently, there is a necessity to develop pilot-scale treatment systems with *E. crassipes* plant biomass material, through adsorption models, in order to identify the optimal parameters for their construction. In view of this, the present project was initiated with the aim of building treatment systems through models using *E. crassipes* biomass with iron chloride.

## 2. Materials and Methods

The utilization of *Eichhornia crassipes* is imperative for this study. The taxonomic classification of this species is (*Eichhornia crassipes*). The collection site is situated within the municipality of Mosquera, situated on the outskirts of Bogotá, D.C., with precise geographical coordinates of 4.682995, −74.256673. The collection comprised 60 dead water hyacinth plants. The *E. crassipes* plants were meticulously washed, with a particular focus on the roots and leaves. The plants were then dried in the sun for three days, after which they were ground until they reached the ideal diameter of 0.212 mm. The pulverized biomass was then sifted through a blade mill.

Chromium measurement: The Cr (VI) content was measured using a UV–Vis spectrophotometer (UV84 Hettich, Föhrenstr, Germany). by monitoring the changes in light absorption. All procedures for the determination of chromium, for water and substrates, were carried out by implementing APHA (American Public Health Association) procedures for standard testing (standard methods for the examination of water and wastewater).

Determination of chromium: The method of diphenylcarbazide was used to estimate the amount of residual chromium (VI). The solution of phosphate buffer was prepared and adjusted to a pH of 2 and a purity level of 90% (H_3_PO_4_). A total of 200 µL of 0.5% diphenylcarbazide (with a purity of 97%), *w*/*v* of acetone with a purity of 97%, 900 µL of phosphate buffer, and 100 µL of the residual sample were placed into an Eppendorf tube. An appropriate portion was then transferred to a cell of absorption, where the absorbancia was measured at 540 nm. The research parameters included initial Cr(VI) concentrations of 400, 600, and 1000 mg/L. Samples were taken at each time interval and the residual chromium concentration was analyzed. Samples of 20 µL were obtained and subsequently taken to the centrifuge (KASAI MIKRO 200). In the present investigation, the tests were conducted under neutral pH conditions, given that the adsorption process is favored in this type of biomass. The study’s measurement uncertainty indicates that heavy element measurements, specifically Cr (VI), can be performed with an uncertainty level of approximately 3.95%.

The desorption–adsorption process: The Cr (VI) adsorption process was followed by an elution process in which the chromium-loaded biomass was washed with distilled water after each cycle of adsorption. The elution process was then carried out in an Erlenmeyer flask at 25 °C for 24 h with constant stirring, using 20 mL of EDTA. The biomass was subsequently separated using a filter [31]. Laboratory-scale elutions utilized 50 milliliters per elution, with a concentration of 3.3 g per liter, while pilot-scale elutions employed a concentration of 3 g per liter, using 300 milliliters per elution [6,7].

Column design and experiments: The objective of this research is to design two distinct treatment systems. The primary system is being developed at the laboratory scale with the intention of facilitating the further development of the secondary system at the pilot scale. The laboratory treatment system has a biomass diameter of 1.5 cm and a biomass length of 22.5 cm. Through the implementation of experimental tests on this system, a larger scale system was developed. The pilot scale treatment system has a diameter of 5 cm with a biomass height of 70 cm. The laboratory scale system contains 45 g.

According to the remaining criteria, the fluid physical properties and linear velocity (v, cm min^−1^) were kept at a constant value in both columns, thus ensuring the same mass transfer and the same hydrodynamic conditions. Initial experiments were performed at the laboratory scale with a flow rate of 20 mL/min, where the flow was managed over the upper capsule, maintaining the system flow rate, ideal treatment conditions in these types of systems [7], with the aim of evaluating three different iron chloride concentrations and biomass densities. The initial Cr(VI) concentration was set at 400, 600, and 1000 mg/L, and the sampling period was set at 50 min. The experiments were performed in a downflow configuration, with three repetitions.

Obtaining iron-modified cellulose: The materials containing iron chloride (Fe_3_ + [FeCl_3_ · 6H_2_O]) [30] were mixed with the biomass of (EC) with the objective of impregnating the reagent in the biomass in dry weight. The mixture was homogenized during the process, where the laboratory scale system contains 45 g of biomass from the various biomasses, thereby creating three design variables.

- 75% (*w*/*w*) *E. crassipes* (33.75 g) and 25% of iron (III) chloride (11.25 g) (EC1)
- 85% (*w*/*w*) *E. crassipes* (38.25 g) and 15% of iron (III) chloride (6.75 g) (EC2)
- 95% (*w*/*w*) *E. crassipes* (42.75 g) and 5% of iron (III) chloride (2.25 g) (EC3)

Development of adsorption models: Equation (1) provides a framework for the conceptualization of treatment systems for eluents laden with heavy metals. The equation is predicated on the premise that the levels preceding and succeeding the contaminant concentrations are known. The initial contaminant concentration, designated as Co, facilitates the calculation of the quantity of contaminant that can be removed at Cf levels within a specified time interval, t, in each volume of water, V, and the surface area, As, occupied by the biomass. The rate of contaminant adsorption is also considered.

(1) $$\frac{\partial C}{\partial t}=k_{F}\times \frac{As}{V}(Co-Cf)$$

(2) $$\frac{\partial C}{\left(Co-Cf\right)}=k_{F}\times \frac{As}{V}\partial t$$

(3) $$Ln\frac{Co}{\left(Cf\right)}=-k_{F}\times \frac{As}{V}t$$

The location is defined by Co and Cf, representing the initial and final concentrations, respectively. The mass transfer coefficient, $k_{F}$, signifies the quantity of contaminant chemisorbed in the biomass per unit of time [31]. V represents the target volume and As is the superficial area.

(4) $$\frac{\partial q}{\partial t}=k_{S}pp\left(qs-q\right)$$

The following formulae are employed: $k_{S}$ is the internal transfer coefficient of the contaminant in the biomass, pp is the density of the microparticle, and qs is the adsorbed capacity in the biomass [32]. In addition, q is the initial capacity in the process (5).

(5) $$q_{S}=\frac{qmBCs}{1+BCs}$$

where B is the Langmuir parameter; if there is a fit to the Langmuir isotherm, this equation must be used to determine the $k_{S}$. If so, utilize Langmuir Equation (6) as follows:

(6) $$ks\left(qs-q\right)=k_{F}\times \frac{As}{V}t$$

Clearing from Equation (7), the constant is Ks.

(7) $$K_{S}=\frac{Kf\times Cs}{Pp(qm\times B-q\times b-q)}+\frac{As\times Kf\times Cs}{V\times Pp(qm\times B-qb-q)}$$

In which $k_{F}$ is the diffusion constant, Cs is the equilibrium concentration (mg/L), pp is the particle density (g/L), qm is the maximum biomass capacity (mg/g), B is the Langmuir isotherm parameter (L/mg), q is the initial capacity, As is the surface area (Cm^2^), and V is the treated volume [31]. Equation (8) is a product equation of mass balances and is used to determine the amount of biomass required to perform adequate treatment, in which the initial concentration, together with the adsorption capacity, plays an important role [31,32].

(8) $$Biomass=\frac{Q\times Tb\times Co}{qm+\frac{\varepsilon \times Co}{Pb}}$$

The parameters of interest are thus Q, the flow rate in (L/min); Tb, the biomass breakthrough time; and Co, the initial concentration in (mg/L). The target volume of the process, denoted V_(target), is defined as a constant and is calculated as follows, V_(target) = Q × Tb, where Q is the flow rate in (L/min), Tb is the biomass breakthrough time, and Co is the initial concentration in (mg/L). The biomass parameter, denoted qm, is used to scale the process. The biomass density, denoted Pb, is also a relevant parameter. The particle 0.0212 mm demonstrated superior performance, likely attributable to its intimate interaction with the contaminant Cr (VI) [6,31]. The relationships between densities ε are between the biomass, in general, and the tiny particle. This relationship is linked to the density of the particle, which must be twice the density of the biomass to ensure a direct relationship with the contaminant. For vegetal biomasses, the ideal value for ε is 0.67 [31]. The surface area As is defined as the volume occupied by the biomass and is used to calculate the coefficient of the relationship of the densities. This coefficient is then used to determine an interesting parameter of scaling. As demonstrated in Equation (9), the surface area is calculated using this process.

(9) $$As=\frac{3\times Vb(1-\varepsilon )}{Rbio}$$

where Vb is the volume occupied by the biomass and Rbio is the radius of the biomass occupied in the treatment system.

## 3. Results

**Figure 1:**
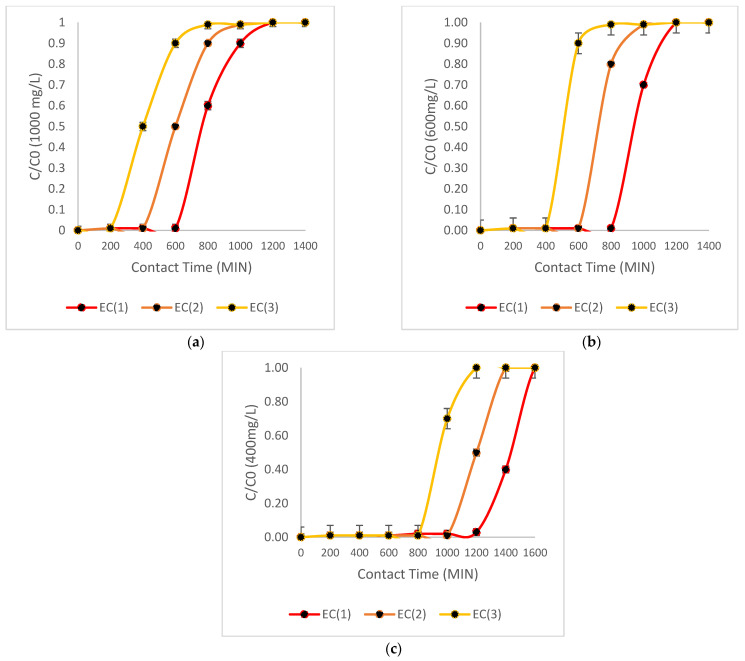
The removal processes by biomass EC with three different concentrations of Cr (VI). (**a**) shows the remotion with 1000 (mg/L) initial, (**b**) shows the remotion with 600 (mg/L) initial, and (**c**) shows the remotion with 400 (mg/L) initial.

Figure 1 illustrates the removal processes of treatment at the laboratory scale by biomass EC with three different concentrations of Cr (VI). The results presented in the graphs are the arithmetic means of the three samples taken in the laboratory.

All biomasses exhibited excellent Cr (VI) ion removal, achieving initial equilibrium levels below those anticipated, likely due to elevated exposure and interaction between the biomasses and the heavy metal. The presence of iron chloride concentrations was shown to enhance active sites, thereby boosting Cr (VI) removal yields. The EC (1) biomass exhibited optimal performance, as anticipated, due to its elevated iron chloride concentration, attaining a superior treatment intensity with a breakpoint of approximately 600 min for elevated initial Cr (VI) concentrations of 1000 mg/L, yielding approximately 4.4 L of treated material. The EC biomass (2) obtained an expected yield of around 3.9 L of water, with a breakpoint of 420 min, while EC (3) removed the Cr (VI) present in 3.4 L of water, with a breakpoint of approximately 390 min. In the presence of cellulose, iron chloride (FeCl_3_) undergoes a progressive oxidation process, resulting in the creation of active sites for the adsorption of heavy metals [51,52]. The removal of metal ion species by Fe is attributable to the oxidation of plant cellulose, thereby facilitating the formation of internal sphere complexes with oxidized sites [53]. In the presence of ECFe biomass, reactions of the (H^+^) of the biomass with the oxygen atoms of the Cr (VI) structure undergo a reduction process, resulting in the formation of Cr (III) and chromium oxide (Cr_2_O_3_). The removal of Cr(VI) by biomaterials is a multifaceted process that involves a combination of adsorption and reduction mechanisms [54,55]. Chlorine reacts with the hydrogen in the biomass, creating (HCl) compounds; this is the reason why this biomass of ECFe tends to be acidic [55]. Utilizing this comprehensive dataset, the mathematical model of the aforementioned Equation (3) was calibrated, with the objective of determining the optimal design parameters for a large-scale model. This includes the ideal treatment concentrations in compliance with regulations, the requisite biomass for effective treatment, and the target volume to be treated. The model establishes and relates the initial and final concentrations, in which the equilibrium point and the treated volume are two of the most significant parameters relating to this adsorption process, as described in Table 1.

The $k_{F}$ value for EC (1) was determined to be 0.6 cm/min, indicating that biomass exhibits superior adsorption speed due to its abundance of active sites and homogeneous adsorption at a higher rate in comparison to the other two biomasses [51,56,57]. However, the $k_{F}$ values for EC (2) and EC (3) were lower, but did not provide conclusive data when making decisions because they obtained ideal treatments and breaking points with promising results. In treatment systems that use plant biomass, bacterial cellulose biomass was observed to reach approximately 1 cm/min [58] and, through the incorporation of various reinforcements, such as Xanthate cellulose, this value can be further augmented. However, data from this study, which recorded a value of 0.45 cm/min [5], fall short of the aforementioned findings. The credibility of research involving heavy metal removal in continuous systems can be enhanced by the objective treatment parameter of both removals and volume. The establishment of the ideal equation to determine the $k_{s}$ coefficient, an ideal complement, is dependent on the behavior of its isotherm.

**Figure 2:**
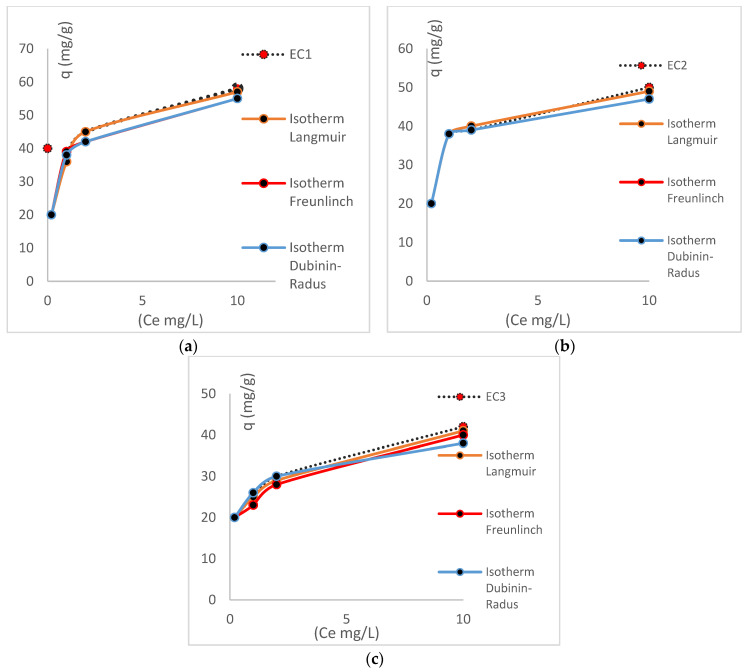
This figure depicts the isotherms of each of the biomasses. (**a**) shows the isotherms of biomass EC1, (**b**) shows the isotherms of biomass EC2, and (**c**) shows the isotherms of biomass EC3.

**Isotherms:** Removal analysis must be performed to establish the isotherms in which Cr(VI) removals by the different biomasses behave. Figure 2 shows the isotherms for each of the biomasses.

As illustrated by the figures above, the isotherm that best relates to the Cr (VI) ion adsorption processes can be established. The Langmuir isotherm demonstrates ideal behavior in the two biomasses of EC (1) and EC (2), due to homogeneity in their adsorption layers, which fill the active sites in an ideal way in the treatment. The biomass EC (3) displays characteristics of both the Freundlich and Langmuir isotherms. The maximum capacity of these biomasses was determined using Equation (7), with the biomass EC (1) achieving an ideal result of 58 mg/g, followed by EC (2) with 50 mg/g and EC (3) with 42 mg/g. The biomass of *E. crassipes* behaved as a homogeneous layer during oxidation with iron chloride, which is consistent with the Langmuir model [59,60,61,62]. Table 2 shows the isotherm representatives.

As Equation (9) is related to the Langmuir isotherm, and due to its significant correlation, this equation was used to determine the intraparticle design variable $k_{s}$ for each biomass, as shown in Table 3. Similarly, the variable $k_{F}$, the constant $k_{s}$, will play a fundamental role in the design and scaling of the treatment system. Table 2 shows the summary of the parameters of the isotherms.

As can be seen in the process, the adsorption capacities that are relevant are those in which the amount of iron chloride predominates. As the amount of iron chloride increases, so too do the yields in the adsorption capacities, due to the fact that iron chloride oxidizes the biomass, creating more active sites [63,64,65]. This parameter will be used to determine the adsorption capacities after each elution.

**Figure 3:**
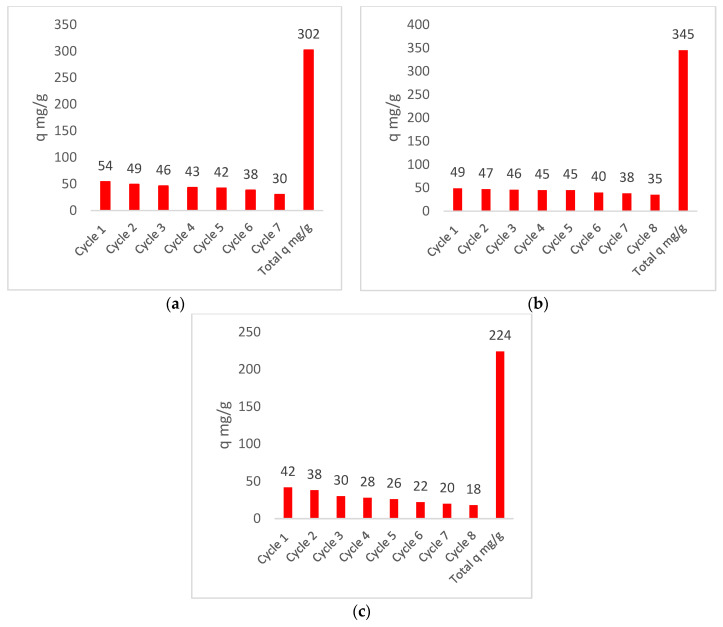
The cycles and reutilizations of biomass. (**a**) shows the elutions of biomass EC1, (**b**) shows the elutions of biomass EC2, and (**c**) shows the elutions of biomass EC3. Table 4 shows each parameters of the processes of the elutions.

EDTA elutions: A key parameter when determining and analyzing the escalation and performance processes is the reuse of the biomass. The *E. crassipes* biomass, containing approximately 20% lignin, displays notable resistance to eluents, withstanding more than five treatment cycles. Figure 3 illustrates the characteristics and elution processes of each biomass, along with their respective removal percentages. Figure 3 shows each process of the elutions.

**Table 4:** The elutions and the behavior of all the biomasses.

Biomass	Elutions	Kf (cm/min)	Volume Goal (L)	Time Break (min)	$k_{s}$ (1/s)	Equation–Isotherm	q_m_
EC (1)	1	0.6	4.4	480	0.017	Langmuir	54
	2	0.58	4.2	460	0.018	Langmuir	49
	3	0.57	3.9	440	0.019	Langmuir	46
	4	0.55	3.7	400	0.020	Langmuir	43
	5	0.45	3.4	370	0.020	Langmuir	42
	6	0.43	3.0	350	0.021	Langmuir	38
	7	0.42	2.4	280	0.021	Langmuir	30
					Total qm		302
EC (2)	1	0.51	4.1	458	0.020	Langmuir	49
	2	0.50	4.0	450	0.021	Langmuir	47
	3	0.45	3.5	410	0.022	Langmuir	46
	4	0.44	3.4	390	0.022	Freundlich	45
	5	0.39	3.3	370	0.020	Langmuir	45
	6	0.38	3.3	350	0.021	Langmuir	40
	7	0.37	3.0	280	0.021	Langmuir	38
	8	0.35	2.8	260	0.020	Langmuir	35
					Total qm		345
EC (3)	1	0.50	3.7	450	0.022	Langmuir	42
	2	0.49	3.2	440	0.023	Langmuir	38
	3	0.48	3.0	380	0.023	Langmuir	30
	4	0.45	2.7	360	0.024	Freundlich	28
	5	0.44	2.2	280	0.020	Langmuir	26
	6	0.40	1.9	240	0.021	Langmuir	22
	7	0.40	1.5	220	0.021	Langmuir	20
	8	0.39	1.5	170	0.020	Langmuir	18
					Total qm		224

As demonstrated in the above table, biomass EC (2) is identified as the optimal choice for the step-up process, owing to the exceptional outcomes observed following elution, as evidenced by the adsorption kinetics and its capacities, which remained unaffected by the EDTA elutions. Biomass EC (1) attained a capacity of 302 mg/g, while biomass EC (2) achieved a capacity of 345 mg/g. Biomass EC (3) obtained 224 mg/g, concluding that the ideal combination between iron chloride and *E. crassipes* biomass is 85% of the vegetal biomass and 15% of the iron chloride. The elutions did not compromise the behavior of their isotherms, for example, as the elutions were carried out, they approached the Freundlich isotherms and moved away from the Langmuir isotherms. This phenomenon can be attributed to the gradual loss of active sites (hydroxyl groups), which concomitantly leads to a reduction in adsorption capacities. Consequently, there is a loss of homogeneity in the biomass, which can be characterized by Freundlich terms. The $k_{s}$ value is determined using the equation developed by Freundlich. Table 5 shows the different experiments with the biomass vegetal.

**Table 5:** The different experiments with the biomass vegetal.

Research	Biomass	Contaminat	q_m_ mg/g	Cicles of Elutions
[66]	Microcrystalline Cellulose core	Cu (II)	423	5
[67]	Aminated Cellulose	Pb (II)	609	5
[68]	Sugarcane Bagasse	Pb (II)	333	3
[69]	Polyacrylicacid/carboxymethyl cellulose/activated carbon	Cu (II)	193	3
[70]	Supramolecular cellulose-based	Co (II)	158	4
[71]	Chitosan/Cellulose-Fe(III)	Cr (VI)	391	6
[72]	β–cyclodextrin modified magnetic cellulose	Pb (II)	200	3
[73]	carboxymethyl cellulose hydrogel	Cu (II)	293	4
[74]	cellulose-derived poly(amidoxime)	Cr (III)	202	3
[75]	Cellulose-ZIF hybrid	Pb (II)	354	4
[76]	Biomass-based aerogel	Cu (II)	380	5
[77]	Alginate-polyvinyl alcohol	Cr (VI)	86	4
[78]	Cellulose Crassipes xantate	Cr (VI)	59	4
[79]	Salvinia molesta	Cr (VI)	33	-
[80]	(*Eichhornia crassipes*) roots	Pb (II)	40	2

It was demonstrated that plant biomasses exhibit intriguing adsorption capacities when subjected to modification, as evidenced in the case of Aminated Cellulose [67], which demonstrated 609 mg/g after five cycles of reuse. In addition, Microcrystalline Cellulose core biomass exhibited 423 mg/g after five cycles [66]. In a separate study, cellulose biomass was modified with iron, resulting in 391 mg/g of Cr (VI) removal capacity [71]. Adsorption capacities exceeding 150 mg/g [68,69,70,71,72,73,74,75,76] indicate the potential for these biomasses to be employed in large-scale processes, as this parameter is a primary consideration in research on bio-adsorbents for contaminant removal. As demonstrated in the research with *E. crassipes,* a plant biomass, these plants have the potential to function as sorbent materials [77,78,79,80].

Cost of treatment systems: *E. crassipes* is not only effective but also very abundant, so its production depends on how close the wetland is to obtain its biomass. The costs were obtained from the unit cost of production of 1 kg. The cost of drying, grinding, and logistics for obtaining *E. crassipes* is approximately USD 2 per kg of this biomass [78]. The cost of iron chloride is USD 0.5 per 100 g [13,81], in addition to an increase of USD 0.5 for all biomasses uniformly for the use of EDTA [43].

It is evident that the EC2 biomass exhibited the optimal indicator, with 116.9 g of Cr (VI) obtained for each dollar invested, attributable to its substantial adsorption capacities of 345 mg/g following each elution process, complemented by a total cost of biomass utilization of USD 2.95. The EC3 biomass demonstrated an indicator of 104 g Cr/(USD), attributable to the relatively low costs, given its comparatively minimal chloride content, in addition to its elevated levels exhibited in the sums of Cr (VI) adsorption capacities (Table 6). This finding suggests that this material could be utilized in scenarios where the allocation of funds for the assembly of effluent treatment systems laden with this heavy metal is limited.

Redesign of process treatment: The redesign of the treatment system was established through the identification of similarities with the process guided by geometric, kinematic, and dynamic similarity criteria [81], relating adsorption capacity (qm) together with its elutions, the amount of biomass and iron chloride and the relationship among densities (ε), the density of the biomass (Pb), and the superficial area (As).

The objective parameters that were identified as a result of this process included the volume of water to be treated (V), the biomass that will treat this contaminated water, and the initial concentration to be treated (Co). Under this scaling process, it was considered that 500 g of biomass would treat 1000 mg/L of initial Cr (VI), in which 550 L of water would be thrown, after seven elutions with EDTA. Equation (8) was utilized, incorporating all available biomass-related data. The scaling parameters are delineated in Table 7.

**Figure 4:**
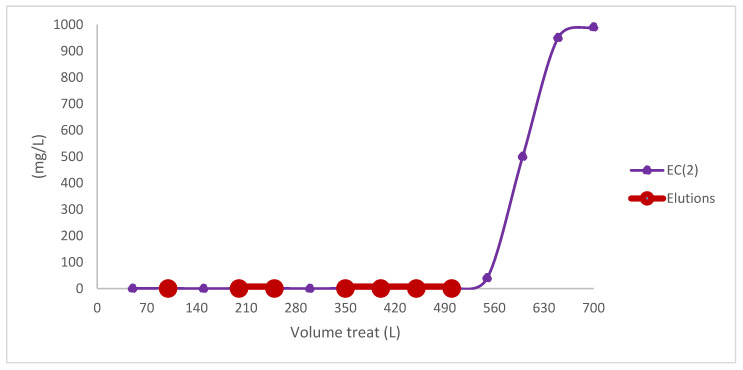
Process of treatment to pilot scale.

**Figure 5:**
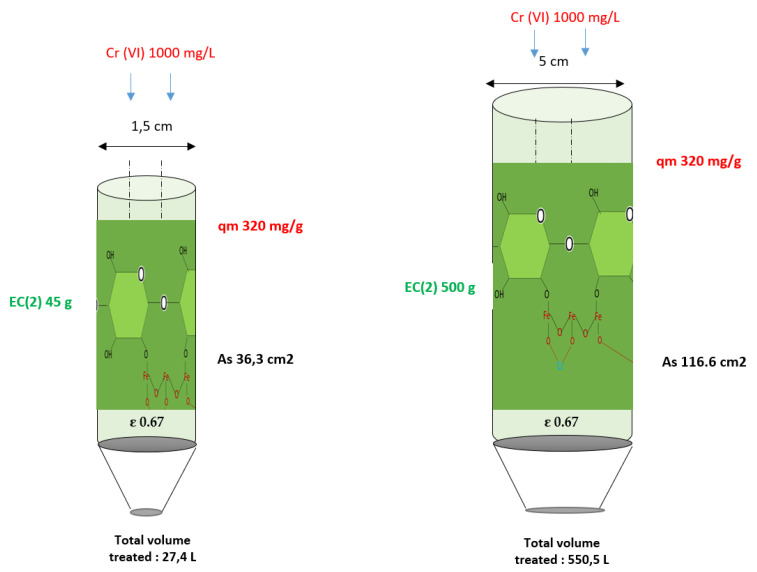
Comparison of scaled systems.

A pilot-scale prototype was constructed, employing 500 g of biomass (425 g of *E. crassipes* 85% and 75 g of iron chloride 15%), analogous to that employed in the laboratory scale. The apparatus consists of one compartment, with a diameter of 5 cm, and EDTA elution processes were incorporated. Figure 4 illustrates the relationship between the treatment systems and their scale-up, while Figure 5 shows the scale-up of both processes.

The laboratory scale model can be seen treating around 27.4 L with a treatment system using 45 g of EC2 biomass in which it has a surface area of 36.3 cm^2^. The pilot scale system was built as recommended by the model with 500 g of biomass, treating 550.5 L of Cr (VI) with an initial 1000 mg/L in which it has 116.6 cm^2^ of surface area, the adsorption capacity is 345 mg/g, but 320 mg/g was used as a margin of error.

Figure 4 shows the treatment of water contaminated with Cr (VI) of 1000 mg/L initially, in which elutions with EDTA were performed when it exceeded 1 mg/L of final Cr (VI) in which the arithmetic average of two procedures is shown.

The pilot-scale apparatus exhibited optimal performance with regard to Cr (VI) removal from water, attaining 99% removal of this heavy metal in the initial treatment processes. In the event of a process failure, the elution process was initiated to reuse the biomass, employing the same methodology as was applied for the laboratory-scale prototype. The experiment involved the treatment of 550 L of water contaminated with Cr (VI). Experiments of this magnitude have been used in the treatment of wastewater with biomass adsorbents, as evidenced in the existing literature [82,83,84,85,86,87,88]. A total of 2.4 L of water containing 3 g/L of EDTA was used in the experiment. This substance was subsequently classified as hazardous waste, as was the residual biomass and the water itself. The Cr (VI) treatment system was found to require an expenditure of approximately USD 1.5 for the treatment of 550 L of water, given the unit cost of USD 3 per kilogram of the biomass used, as outlined in the preceding section. For instance, in order to treat 1 m^3^ of water contaminated with initial Cr (VI) and employing the same initial design parameters, a treatment system comprising 911 g of EC biomass would be required. This system would have a unit production cost of approximately USD 5, which includes the elutions and final disposals in addition to the device. The device is notable for being one of the most economical devices with heavy metal adsorbent material in the current literature [89,90,91]. In addition to being economical, it is also easy to install and very effective in removing these contaminants in the water, compared to those developed in current research in the literature.

## 4. Conclusions

The Cr (VI) removal treatment process at the laboratory scale, using three different types of *E. crassipes* plant biomass with variable iron chloride concentrations of 25% EC (1), 15% EC (2), and 5% EC (3), was carried out with the aim of establishing the adsorption models that would determine the ideal parameters for the process at the laboratory scale. The process was scaled up with the most effective biomass for treatment and subsequent elutions with EDTA. The outlet conditions were established, i.e., the amount of pollutant removed and the treated volume, determining the extraparticle diffusion constant, $k_{F}$, subsequently the intraparticle diffusion constant and the characteristic isotherm were determined, identifying the intraparticle diffusion model, $k_{s}$. The results of the model indicated the specific route for the construction of a pilot scale treatment system. EC (1) is ideal for Cr (VI) removal. However, EC (2) has a better accumulated adsorption capacity and therefore a better cost index g Cr/(USD). The pilot-scale prototype was constructed using 1 kg of the EC (2) biomass, which exhibited the optimal blend of *E. crassipes* biomass and iron chloride. This combination demonstrated superior removal performance and resilience in the EDTA elution process, which costs around USD 3, making it very economical compared to conventional heavy metal treatment systems. The simplicity of the adsorbent preparation, its reusability, and its adsorption capacity suggest that *E. crassipes* biomass, in combination with iron chloride and the EDTA elution process, has the potential to be applied for the removal of various Cr (VI)-laden effluents. The development of a prototype on a pilot scale is instrumental in establishing a fundamental basis of sustainable development, thereby ensuring the attainment of desired access to clean and safe water. This, in turn, contributes to enhancing public health, environmental protection, and economic growth, the rationale underpinning the selection of this particular device is threefold: firstly, its low cost; secondly, the ease with which it can be installed; thirdly, the preparation of the biomass; and finally, its efficiency in terms of treatment.

## Figures and Tables

**Table 1:** Summary of parameters given.

Biomass	As cm^2^	$k_{F}$ (cm/min)	Volume Goal (L)	Time Break (min)
EC (1)	36.3	0.6	4.4	480
EC (2)	36.3	0.50	3.9	420
EC (3)	36.3	0.45	3.4	390

**Table 2:** Isotherm representatives.

	Isotherm	Parameters	R^2^
EC (1)	Langmuir	B = 0.6; q_m_; 58	0.99
	Freundlich	K = 0.17	0.91
	Dubinin Radus	B = 0.017; q_m_ 55	0.89
	Isotherm	Parameters	R^2^
EC (2)	Langmuir	B = 0.5; q_m_; 50	0.99
	Freundlich	K = 0,11	0.92
	Dubinin Radus	B = 0.018; q_m_ 49	0.95
	Isotherm	Parameters	R^2^
EC (3)	Langmuir	B = 0.4; q_m_; 42	0.98
	Freundlich	K = 0.10	0.96
	Dubinin Radus	B = 0.019; q_m_ 40	0.90

**Table 3:** Isotherm equation representatives.

Biomass	$k_{s}$ (1/s)	Equation–Isotherm	q_m_
EC (1)	0.016	Langmuir	58
EC (2)	0.017	Langmuir	50
EC (3)	0.019	Langmuir	42

**Table 6:** Biomass costs compared to adsorption capacities.

Cost	EC1	EC2	EC3
Capacity total (g Cr/kg material)	302	345	224
Cost (USD) 1 Kg material	3.25	3.0	2.15
g Cr/(USD)	92.92	116.9	104.18

**Table 7:** Parameters of scaling.

	M (g)	Volume Mass (Vb)	Density of Biomass	As Cm^2^	Caudal mL/min	Volume Treat (L)	Elutions
EC (2)	45	55	0.85	61.5	20	27.4	8
Scalling	500	583	0.85	116.6	200	550.5	7

## Data Availability

The data presented in this study are available on request from the author.
